# The Neuroprotective Effects of Brazilian Green Propolis on Neurodegenerative Damage in Human Neuronal SH-SY5Y Cells

**DOI:** 10.1155/2017/7984327

**Published:** 2017-02-06

**Authors:** Junjun Ni, Zhou Wu, Jie Meng, Aiqin Zhu, Xin Zhong, Shizheng Wu, Hiroshi Nakanishi

**Affiliations:** ^1^Department of Aging Science and Pharmacology, Faculty of Dental Science, Kyushu University, Fukuoka 812-8582, Japan; ^2^OBT Research Center, Faculty of Dental Science, Kyushu University, Fukuoka 812-8582, Japan; ^3^Institute of Geriatrics, Qinghai Provincial Hospital, Xining 810007, China

## Abstract

Oxidative stress and synapse dysfunction are the major neurodegenerative damage correlated to cognitive impairment in Alzheimer's disease (AD). We have found that Brazilian green propolis (propolis) improves the cognitive functions of mild cognitive impairment patients living at high altitude; however, mechanism underlying the effects of propolis is unknown. In the present study, we investigated the effects of propolis on oxidative stress, expression of brain-derived neurotrophic factor (BDNF), and activity-regulated cytoskeleton-associated protein (Arc), the critical factors of synapse efficacy, using human neuroblastoma SH-SY5Y cells. Pretreatment with propolis significantly ameliorated the hydrogen peroxide- (H_2_O_2_-) induced cytotoxicity in SH-SY5Y cells. Furthermore, propolis significantly reduced the H_2_O_2_-generated reactive oxygen species (ROS) derived from mitochondria and 8-oxo-2′-deoxyguanosine (8-oxo-dG, the DNA oxidative damage marker) but significantly reversed the fibrillar *β*-amyloid and IL-1*β*-impaired BDNF-induced Arc expression in SH-SY5Y cells. Furthermore, propolis significantly upregulated BDNF mRNA expression in time- and dose-dependent manners. In addition, propolis induced Arc mRNA and protein expression via phosphoinositide-3 kinase (PI3K). These observations strongly suggest that propolis protects from the neurodegenerative damage in neurons through the properties of various antioxidants. The present study provides a potential molecular mechanism of Brazilian green propolis in prevention of cognitive impairment in AD as well as aging.

## 1. Introduction

Alzheimer's disease (AD) is the most common form of dementia in aging societies worldwide [1] and the number of AD is growing dramatically [2]. Oxidative stress is a major component of the harmful cascades activated in the development of aging-related neurodegenerative disorders, including AD [3, 4], because the overproduction of reactive oxygen species (ROS) causes cell damage through the promotion of lipid peroxidation, DNA damage, and the regulation of death proteins [5]. Antioxidant therapy therefore is considered as an approach in prevention and clinical management of AD [6]. On the other hand, dysfunction of hippocampal synaptic efficacy is known to be related to cognitive impairment in AD [7, 8] as well as in aging [9–11], and synaptic efficacy has protective effects against AD [12, 13].

Activity-regulated cytoskeleton-associated protein (Arc) is a critical immediate-early gene that has been implicated in generated stable changes in synaptic efficacy [14, 15], as suppression of Arc expression impairs the synaptic plasticity and memory consolidation [11, 14, 16]. As a member of the neurotrophin family, brain-derived neurotrophic factor (BDNF) plays a fundamental role in the synaptic efficacy by inducing Arc expression [17, 18], and BDNF-induced Arc expression contributes to cognitive functions [14, 19, 20]. Indeed, fibrillar *β*-amyloid, the main component of plaques in the brains of AD patients, has been shown to impair the BDNF-induced Arc expression in the cultured cortical neurons, even at low levels [21]; therefore, interfering with BDNF signaling affects the downstream neuronal functions that contribute to the development of AD [22]. However, mounting evidence suggests that neuroinflammation, induced by proinflammatory cytokines, increases the risk of cognitive impairment [23–25]. As a potent activator for exacerbating neuroinflammation, interleukin-1*β* (IL-1*β*) has been shown to mediate synaptic efficacy by suppressing BDNF-induced Arc expression [26].

Propolis is a resinous substance produced by honeybees as a defense against intruders. It has relevant therapeutic properties that have been used since ancient times. The chemical composition of propolis depends on the local floral at the site of collection [27, 28]. The neuroprotective effects of propolis and its active components, such as baccharin, p-Coumaric acid, and Artepillin C, have been established [29–32]. In addition, Artepillin C, a major component of Brazilian propolis, has been shown to act as a neurotrophic-like factor for promoting neuron growth factor- (NGF-) induced neurite outgrowth [33]. Our ongoing human research at high altitude has shown that elderly individuals treated with propolis score significantly higher on cognitive tests than nontreated subjects (Zhu and Wu unpublished data), and we hypothesized that propolis may have neuroprotective effects on neurons directly. However, we recently found that propolis protects against the hypoxia-induced microglia mediated neuroinflammation [34, 35]. In the present study, we focused on the effects of propolis on the hydrogen peroxide- (H_2_O_2_-) induced oxidative stress, and expression of BDNF and Arc using cultured human neuroblastoma SH-SY5Y cells, which are widely used for the study of neurodegenerative damage in vitro [36].

## 2. Material and Methods

### 2.1. Reagents

Brazilian green propolis ethanol extract (propolis) was purchased from Yamada Apiculture Center, Inc. (Okayama, Japan). Minimal essential medium (MEM), F12, fetal bovine serum (FBS), penicillin-streptomycin. and Hoechst 33342 were purchased from Thermo Fisher Scientific (Waltham, MA, USA). H_2_O_2_ (30%), GF 109203X hydrochloride (protein kinase C inhibitor), and ANA-12 (TrkB selective antagonist) were purchased from Selleckchem (Houston, TX, USA). Bay11-7082 (a specific NF-*κ*B inhibitor), U0126 (ERK inhibitors), Carbachol, and human BDNF were purchased from Sigma-Aldrich Inc. (St. Louis, MO, USA). Y-27632 dihydrochloride (Rho-associated protein kinase inhibitor) was purchased from TOCRIS (Avonmouth, Bristol, UK). Wortmannin (phosphoinositide 3-kinase inhibitor) was purchased from Millipore (California, USA). Artepillin C was purchased from WAKO (Osaka, Japan). RNAiso Plus was purchased from Takara (Hoto-ku, Osaka, Japan). QuantiTect Reverse Transcription Kit and Rotor-Gene SYBR Green RT-PCR Kit were purchased from Qiagen (Hilden, Germany). A Cell-Counting Kit (CCK-8) was purchased from Dojindo (Kumamoto, Japan). Human recombinant IL-1*β* was purchased from R&D (Minneapolis, USA). *β*-Amyloid (1–42) was purchased from ANASPEC (California, USA), dissolved in endotoxin-free sterile water and incubated at 37°C for 24 h to induce the fibril formation.

### 2.2. SH-SY5Y Cell Culture

Human neuroblastoma SH-SY5Y cells purchased from American Type Culture Collection (Manassas, VA, USA) were maintained in a complete growth medium (MEM/F-12 mixture containing 10% fetal bovine serum, supplemented with NaHCO_3_ and 100 U/mL penicillin-streptomycin). The cells were cultured at 37°C in a 5% CO_2_ humidified incubator.

### 2.3. Cell Viability Assay

SH-SY5Y cells were seeded in 96-well plates (5 × 10^3^ cells/well) overnight. After treatment with propolis, fA*β*, and IL-1*β*, a cell viability assay was performed using a Cell-Counting Kit (Dojindo) in accordance with the previously described method [34]. The optical density was read at a wavelength of 450 nm with a microplate reader. The cell viability was calculated by dividing the optical density of the treated group by that of the control group.

### 2.4. Observations of Morphological Changes

The cells were seeded in 6-well plates (2 × 10^5^ cells/mL) for 24 h and then treated with H_2_O_2_ at a concentration of 100 *μ*M for 24 h with or without propolis (methanol extraction). The cellular morphology was observed and photographed using a bright-field microscope (Nikon, ECLIPSE Ti-S, Japan).

### 2.5. Detection of Mitochondrial ROS

Mitochondrial ROS were measured using MitoSOX™ Red (Invitrogen, USA), which is a live-cell permeant that rapidly and selectively targets mitochondria [37]. Once in the mitochondria, MitoSOX Red reagent is oxidized by superoxide and exhibits red fluorescence (with excitation at 510 nm and emission at 580 nm). The cultured SH-SY5Y cells were seeded in 24-well plates (2 × 10^5^ cells/mL) and incubated with or without propolis for 1 h (methanol extraction, 10 *μ*g/mL). The cells were then further exposed to H_2_O_2_ (100 *μ*M) for 1 h. After incubation in Hank's balanced salt solution (HBSS) containing 5 mM MitoSOX Red for 10 min at 37°C, the cells were washed twice with PBS and then mounted in a warm buffer for imaging. Images were collected using a fluorescence microscope (Nikon, ECLIPSE Ti-S, Japan).

### 2.6. Immunofluorescence Imaging

Immunofluorescence imaging was performed as described previously [34, 38]. SH-SY5Y cells were seeded in 24-well plates (2 × 10^5^ cells/mL) for 24 h and then treated with H_2_O_2_ (100 *μ*M) for 4 h, with or without pretreatment with propolis (methanol extraction, 10 *μ*g/mL) for 1 h, and fixed with 4% paraformaldehyde. After washing the cells with PBS twice, they were incubated with mouse anti-8-oxo-dG (1 : 500) overnight at 4°C and then incubated with anti-mouse Alexa 488 (1 : 2000, Jackson Immunoresearch Lab. Inc.) at 4°C for 2 h. After washing by PBS, the nucleus was stained by Hoechst 33342. The cells were mounted in the antifading medium, Vecta shield, and the fluorescence images were taken using a confocal laser scanning microscope (CLSM; C2si, Nikon, Japan).

### 2.7. Real-Time Quantitative Polymerase Chain Reaction (qRT-PCR)

The mRNA isolated from the SH-SY5Y cells at various time points were subjected to a real-time qRT-PCR. The total RNA was extracted using the RNAiso Plus in accordance with the manufacturer's instructions. A total of 800 ng of extracted RNA was reverse transcribed to cDNA using the QuantiTect Reverse Transcription Kit. After an initial denaturation step at 95°C for 5 min, temperature cycling was initiated. Each cycle consisted of denaturation at 95°C for 5 s, annealing at 60°C for 10 s, and elongation for 30 s. In total, 40 cycles were performed. The cDNA was amplified in duplicate using a Rotor-Gene SYBR Green RT-PCR Kit with a Corbett Rotor-Gene RG-3000A Real-Time PCR System (Sydney, Australia). The data were evaluated using the RG-3000A software program (version Rotor-Gene 6.1.93, Corbett). The sequences of primer pairs were as follows: Arc, 5′-CCACCTGCTTGGACACCTC-3′ and 5′-CCGCCCCGAGGAGTTTG-3′; BDNF, 5′-GGATGAGGACCAGAAAGT-3′ and 5′-AGCAGAAAGAGAAGAGGAG-3′; actin, 5′-AGAGCTACGAGCTGCCTGAC-3′ and 5′-AGCACTGTGTTGGCGTACAG-3′.

For data normalization, an endogenous control (actin) was assessed to control for the cDNA input, and the relative units were calculated by a comparative Ct method. All of the real-time qRT-PCR experiments were repeated three times, and the results are presented as the means of the ratios ± standard error of the mean.

### 2.8. Electrophoresis and Immunoblotting

SH-SY5Y cells were cultured at a density of 2 × 10^5^ cells/mL. Cells were harvested at each time point with various stimulations, and immunoblotting analyses were conducted. Briefly, each specimen was electrophoresed using 12% SDS-polyacrylamide gels. The proteins on the SDS gels were then electrophoretically transferred to nitrocellulose membranes. Following blocking, the membranes were incubated at 4°C overnight under gentle agitation with each primary antibody: mouse anti-Arc (1 : 1000; Abcam, Heidelberg, Germany) and mouse anti-actin (1 : 5000; Abcam). After washing, the membranes were incubated with horseradish peroxidase- (HRP-) labeled anti-mouse (1 : 2000; GE Healthcare, Buckinghamshire, UK) for 2 h at room temperature. Subsequently, the membrane-bound, HRP-labeled antibodies were detected using an enhanced chemiluminescence detection system (ECK lit; GE Healthcare) with an image analyzer (LAS-1000; Fuji Photo Film; Minato-ku, Tokyo, Japan).

### 2.9. Statistical Analysis

The data are represented as the means ± standard error of the mean. The statistical analyses were performed by a one- or two-way analysis of variance with a post hoc Tukey's test using the GraphPad Prism software package (GraphPad Software, California, USA). A value of *p* < 0.05 was considered to indicate statistical significance.

## 3. Results

### 3.1. Effect of Propolis on Cell Viability in SH-SY5Y Cells

**Figure 1:**
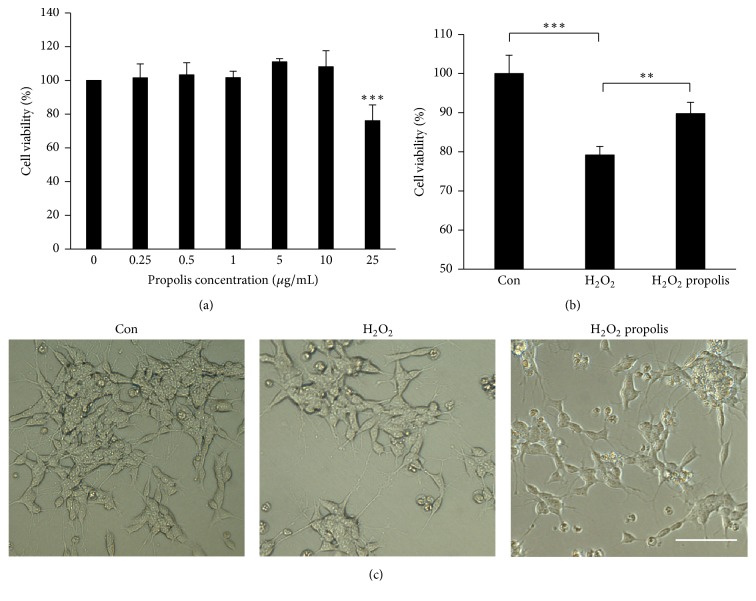
Effects of methanol extracts of propolis on the H_2_O_2_-induced toxicity in SH-SY5Y cells. (a) Cell viability in SH-SY5Y cells pretreatment with different concentrations of propolis for 48 hours. (b) The effect of pretreatment with methanol extracts of propolis for 2 h on H_2_O_2_ exposed SH-SY5Y cells. Each column and bar represent mean ± SEM (*n* = 4 each). An asterisk indicates a statistically significant difference from the indicated group value (^*∗∗*^*p* < 0.01, ^*∗∗∗*^*p* < 0.001). (c) The morphological changes of SH-SY5Y cells with or without pretreatment with propolis (10 *μ*g/mL) after expose to H_2_O_2_ for 24 h. Scale bar = 20 *μ*m.

We first examined the effects of propolis on the viability of SH-SY5Y cells using CCK8 assay kit. The mean cell viability was not significantly changed after treatment with the ethanol extracts of propolis at final concentrations between 0.25 and 10 *μ*g/mL for 48 h (Figure 1(a)). However, the mean cell viability was significantly reduced after pretreatment with propolis at a final concentration of over 25 *μ*g/mL (75% of viable cells). We therefore used methanol extracts of propolis at concentrations up to 10 *μ*g/mL and H_2_O_2_ at 100 *μ*M concentration [38] to investigate the effects of propolis on the H_2_O_2_-induced cytotoxicity of SH-SY5Y cells in the subsequent experiments. The H_2_O_2_-induced cell death (79% of viable cells) was significantly restored by pretreatment with propolis (90% of viable cells) (Figure 1(b)). Morphological changes observed included the degeneration of H_2_O_2_-treated SH-SY5Y cells, which exhibited the disappearance of the neurites and shrinkage (Figure 1(c)). The percentage of viable cells was also reduced by H_2_O_2_ in a dose-dependent manner (data not shown). It is noted that the neuritis and shrinkage of cells were attenuated by pretreatment with propolis (Figure 1(c)). These observations strongly demonstrate that pretreatment with propolis protects SH-SY5Y cells from H_2_O_2_-induced cytotoxicity. Pretreatment with methanol extracts of propolis (10 *μ*g/mL) for 1 h was set up in the subsequent experiments.

### 3.2. Effects of Propolis on the H_2_O_2_-Induced Oxidative Stress in SH-SY5Y Cells

**Figure 2:**
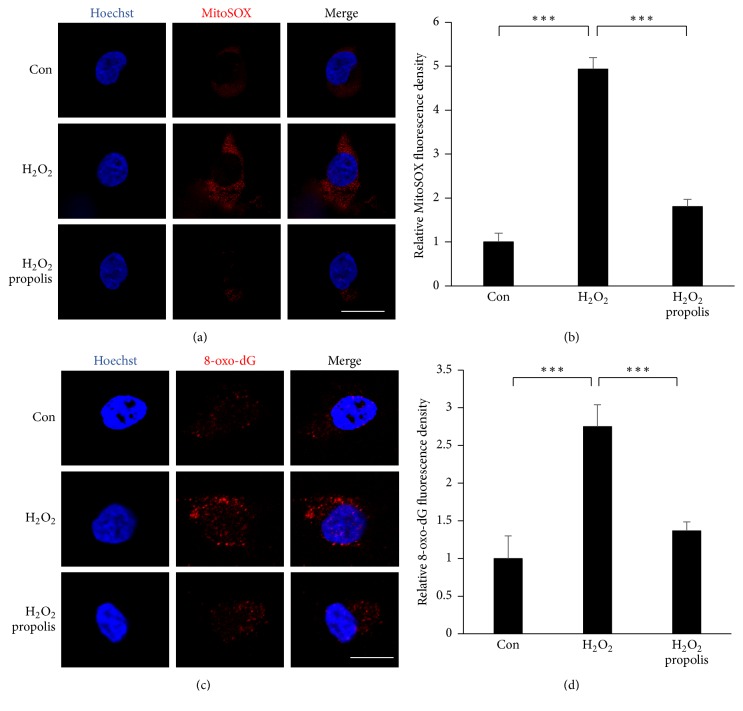
Effects of methanol extracts of propolis on the H_2_O_2_-induced oxidative stress in SH-SY5Y cells. (a) Fluorescent images of MitoSOX Red signals in SH-SY5Y cells exposed to H_2_O_2_ for 1 h with or without propolis (10 *μ*g/mL) for 2 h. Scale bar = 15 *μ*m. (b) The quantitative analyses of MitoSOX Red signal intensity in (a). (c) Immunofluorescent CLMS images of 8-oxo-dG (red) with Hoechst-stained nuclei (blue) in SH-SY5Y cells exposed to 100 *μ*M of H_2_O_2_ for 4 h with or without pretreatment with propolis (10 *μ*g/mL) for 2 h. Scale bar = 10 *μ*m. (d) The quantitative analyses of 8-oxo-dG immunofluorescence signal intensity in (c). The results are expressed as the mean ± SEM (*n* = 4 each), and the asterisks indicate a statistically significant difference from the indicated group value (^*∗∗∗*^*p* < 0.001).

Oxidative stress is an important inducer of neurotoxicity in AD patients [39]; following our previous experiments, we used two approaches to address the effects of propolis on H_2_O_2_-induced oxidative stress in SH-SY5Y cells: one approach was the use of a MitoSOX Red probe, as a marker for mitochondria-derived ROS generation [35], and the other was immunofluorescence imaging for a biomarker of oxidation-damaged DNA marker, 8-oxo-dG [40]. In comparison to the untreated cells, the expression of MitoSOX Red signals was significantly increased in SH-SY5Y cells after exposure to H_2_O_2_ for 1 h, suggesting that the mitochondria are the early origin of ROS generation during oxidative stress. Pretreatment with propolis significantly inhibited the H_2_O_2_-induced mitochondria-derived ROS generation in SH-SY5Y cells (Figure 2(a)), and the mean fluorescent intensity of MitoSOX Red oxidation was found to significantly increase in comparison to that in the cells that were not exposed to H_2_O_2_ (4.93 versus 1, ^*∗∗∗*^*p* < 0.001, Figure 2(b)). Pretreatment with the propolis methanol extracts for 1 h significantly reduced the immunofluorescence intensity of MitoSOX Red oxidation in the H_2_O_2_-exposed SH-SY5Y cells (1.8 versus 4.93, ^*∗∗∗*^*p* < 0.001, Figure 2(b)), thus confirming the antioxidant properties of propolis. Immunofluorescence imaging showed a significant inverse relationship between Hoechst and 8-oxo-dG after exposure of SH-SY5Y cells to H_2_O_2_ for 4 h (Figure 2(c)), and the mean fluorescent intensity of 8-oxo-dG was found to significantly increase in comparison to that in the cells that were not exposed to H_2_O_2_ (2.75 versus 1, ^*∗∗∗*^*p* < 0.001, Figure 2(d)). It is noted that pretreatment with the propolis for 2 h significantly reduced the immunofluorescence intensity of 8-oxo-dG in the H_2_O_2_-exposed SH-SY5Y cells (1.36 versus 2.75, ^*∗∗∗*^*p* < 0.001, Figure 2(d)). These observations demonstrate that propolis could attenuate oxidative stress in neuronal cells.

### 3.3. Effect of Propolis on the Fibrillar A*β*-Induced Impairment of BDNF-Induced Arc Expression in SH-SY5Y Cells

**Figure 3:**
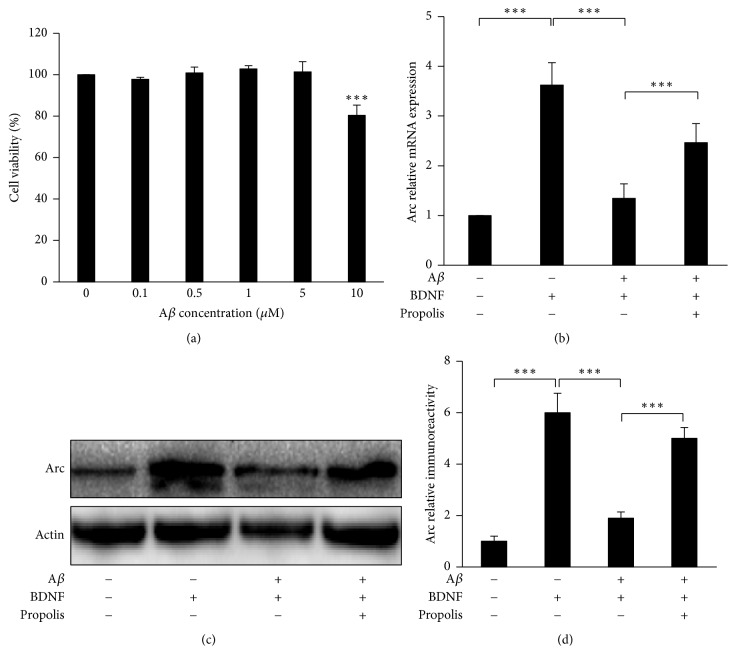
The effect of methanol extracts of propolis on the A*β*-induced impairment of BDNF-induced Arc expression in SH-SY5Y cells. (a) SH-SY5Y cells were treated with the indicated concentrations of propolis for 48 h. The cell viability of SH-SY5Y was then measured using a CCK-8 Assay Kit. (b) SH-SY5Y cells were treated with indicated concentrations of fA*β* for 48 h. The cell viability of SH-SY5Y was then measured again using a CCK-8 Assay Kit. (c) SH-SY5Y cells were pretreated with fA*β* (5 *μ*M) for 6 h, followed by incubation with BDNF (10 ng/mL) for 2 h. Propolis was treated 2 h before BDNF application. (d) The expression of Arc after treatment with fA*β*, propolis, or BDNF. (d) The quantitative analysis of Arc protein expression. The results are expressed as the mean ± SEM (*n* = 4 each), and the asterisks indicate a statistically significant difference from the indicated group value (^*∗∗∗*^*p* < 0.001).

Soluble A*β* is known to interfere with synaptic efficacy, as cognitive decline precedes the formation of A*β* plaques, the hallmark of AD [41]. We therefore examined the effects of propolis on the impairment of BDNF-induced Arc expression by fA*β* in SH-SY5Y cells, because Arc is the critical factor of synapse efficacy. The mean cell viability was not significantly changed after treatment with fA*β* at final concentrations between 0.1 and 5 *μ*M for 48 h (Figure 3(a)). However, significant cytotoxicity was observed in cultures treated with fA*β* over 10 *μ*M (80% of viable cells). Therefore, 5 *μ*M was used as the nonlethal concentration for SH-SY5Y cells in subsequent experiments. Treatment with BDNF (10 ng/mL) for 120 min induced 3.5-fold Arc expression in SH-SY5Y cells; however, preexposure to fA*β* (5 *μ*M) for 6 h suppressed the BDNF-induced Arc expression (Figure 3(b)). Interestingly, this impairment was significantly reversed following treatment with propolis (10 *μ*g/mL) for 120 min (Figure 3(b)). Furthermore, propolis treatment also prevented the fA*β*-induced impairment of BDNF-induced Arc protein expression (Figures 3(c) and 3(d)). These data indicate that propolis reverses the fA*β*-induced impairment of BDNF-induced Arc mRNA and protein expression.

### 3.4. Effect of Propolis on the IL-1*β*-Induced Impairment of BDNF-Induced Arc Expression in SH-SY5Y Cells

**Figure 4:**
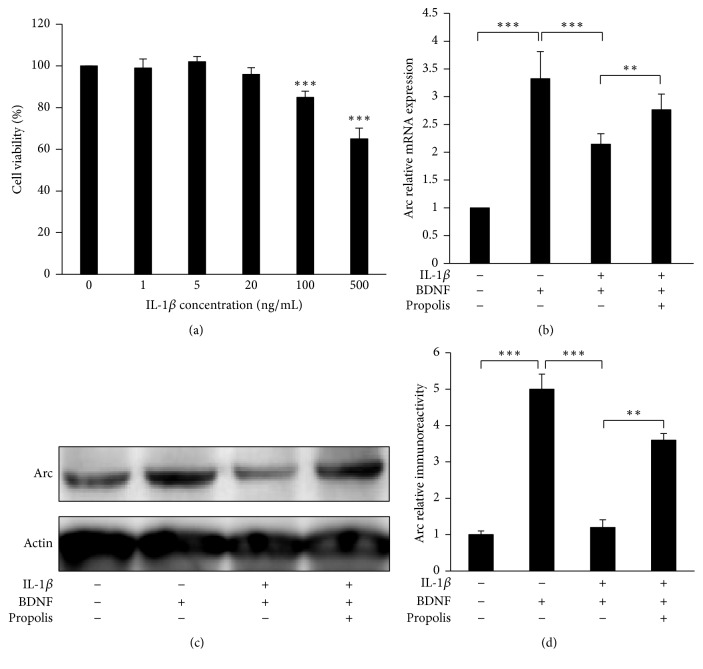
The effect of methanol extracts of propolis on the IL-1*β*-induced impairment of BDNF-induced Arc expression in SH-SY5Y cells. (a) SH-SY5Y cells were treated with the indicated concentrations of IL-1*β* for 48 h. The cell viability of SH-SY5Y was then measured using a CCK-8 Assay Kit. (b) SH-SY5Y cells were pretreated with IL-1*β* (20 ng/mL) for 6 h followed by incubation with BDNF (10 ng/mL) for 2 h. Propolis was treated 2 h before BDNF application. (c) The expression of Arc after treatment with IL-1*β*, propolis, or BDNF. (d) The quantitative analysis of Arc protein expression. The results are expressed as the mean ± SEM (*n* = 4 each), and the asterisks indicate a statistically significant difference from the indicated group value (^*∗∗*^*p* < 0.01, ^*∗∗∗*^*p* < 0.001).

As a potent activator for exacerbating neuroinflammation, IL-1*β* has recently been found to suppress BDNF-dependent synaptic efficacy [26]. We therefore examined the effects of propolis on the IL-1*β*-induced impairment of BDNF-induced Arc expression in SH-SY5Y cells. We first examined the effects of IL-1*β* on the cell viability to determine the nonlethal concentration of IL-1*β* on SH-SY5Y cells. The mean cell viability was not significantly changed after treatment with IL-1*β* at final concentrations between 1 and 500 ng/mL for 48 h (Figure 4(a)). However, significant cytotoxicity was observed in cultures treated with IL-1*β* over 100 ng/mL (90% of viable cells). We therefore used 20 ng/mL as the nonlethal concentration for SH-SY5Y cells in subsequent experiments.

Treatment with BDNF (10 ng/mL) for 120 min induced 3.4-fold Arc expression in SH-SY5Y cells; however, preexposure to IL-1*β* (20 ng/mL) for 6 h significantly suppressed the BDNF-induced Arc expression (Figure 4(b)). Interestingly, this impairment was significantly reversed following treatment with propolis (10 mg/mL) for 120 min (Figure 4(b)). Furthermore, propolis treatment also prevented the IL-1*β*-induced impairment of BDNF-induced Arc protein expression (Figures 4(c) and 4(d)). These data indicate that propolis reverses the IL-1*β*-induced impairment of BDNF-induced Arc mRNA and protein expression.

### 3.5. Effect of Propolis on BDNF Expression in SH-SY5Y Cells

**Figure 5:**
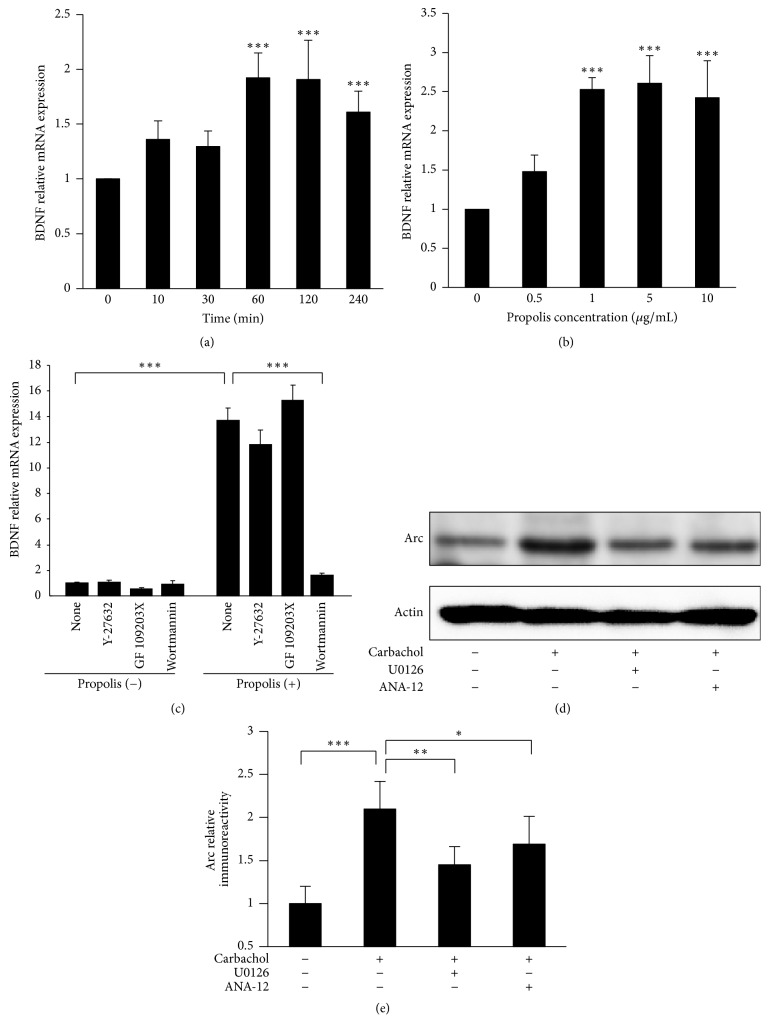
The effect of methanol extracts of propolis on BDNF expression in SH-SY5Y cells. (a) Time-dependent effects of 10 *μ*g/mL propolis on BDNF mRNA expression in SH-SY5Y cells. (b) Dose-dependent effects of propolis on Arc mRNA expression in SH-SY5Y cells at 60 min after incubation. (c) Effects of specific inhibitors on BDNF mRNA expression. GF109203X hydrochloride (protein kinase C inhibitor, 200 *μ*M), Y-27632 dihydrochloride (Rho-associated protein kinase inhibitor, 1 *μ*M), and Wortmannin (phosphoinositide 3-kinase inhibitor, 200 nM) were pretreated for 1 h, followed by incubation with 10 *μ*g/mL propolis for 30 min. (d) The expression of Arc after treatment with Carbachol (1 mM) and pretreatment with U0126 (ERK inhibitors, 10 *μ*M) or ANA-12 (TrkB selective antagonist, 100 nM). (e) The quantitative analysis of Arc protein expression shown in (d). The results are expressed as the mean ± SEM (*n* = 4 each), and the asterisks indicate a statistically significant difference from the indicated value (^*∗*^*p* < 0.01, ^*∗∗*^*p* < 0.005, and ^*∗∗∗*^*p* < 0.001).

We next examined the effects of propolis on BDNF expression in SH-SY5Y cells, because BDNF is the critical neurotrophic factor that is important for synapse efficacy. Pretreatment with propolis significantly increased the BDNF mRNA expression in SH-SY5Y cells from 60 min up to 240 min, even at a low concentration (1 *μ*g/mL) (Figure 5(a)). Furthermore, treatment with propolis for 60 min increased the BDNF mRNA expression in SH-SY5Y cells in a dose-dependent manner (Figure 5(b)). The propolis-increased BNDF expression was completely abolished by preincubation with Wortmannin (200 nM, PI-3K inhibitor), but not by treatment with Y27632 (1 *μ*M, ROCK inhibitor) or GFX (200 nM, PKC inhibitor) (Figure 5(c)). BDNF/TrkB signaling has been reported to play an important role in long term potential to further confirm the BDNF signaling to Arc, we use a Carbachol to stimulate SH-SY5Y, and the responses of Carbachol were examined in the presence of U0126 (ERK inhibitors, 10 *μ*M) or ANA-12 (TrkB selective antagonist, 100 nM).

1 h of treatment with Carbachol can significantly increase the protein expression level of Arc. However, pretreatment with U0126 and ANA significantly reduced the Carbachol-increased Arc (Figures 5(d) and 5(e))). Therefore, the BDNF/TrkB/ERK may regulate the Arc in SH-SY5Y. These observations demonstrate that propolis upregulates BNDF expression through PI-3K-dependent pathways.

### 3.6. Effect of Propolis on Arc Expression in SH-SY5Y Cells

**Figure 6:**
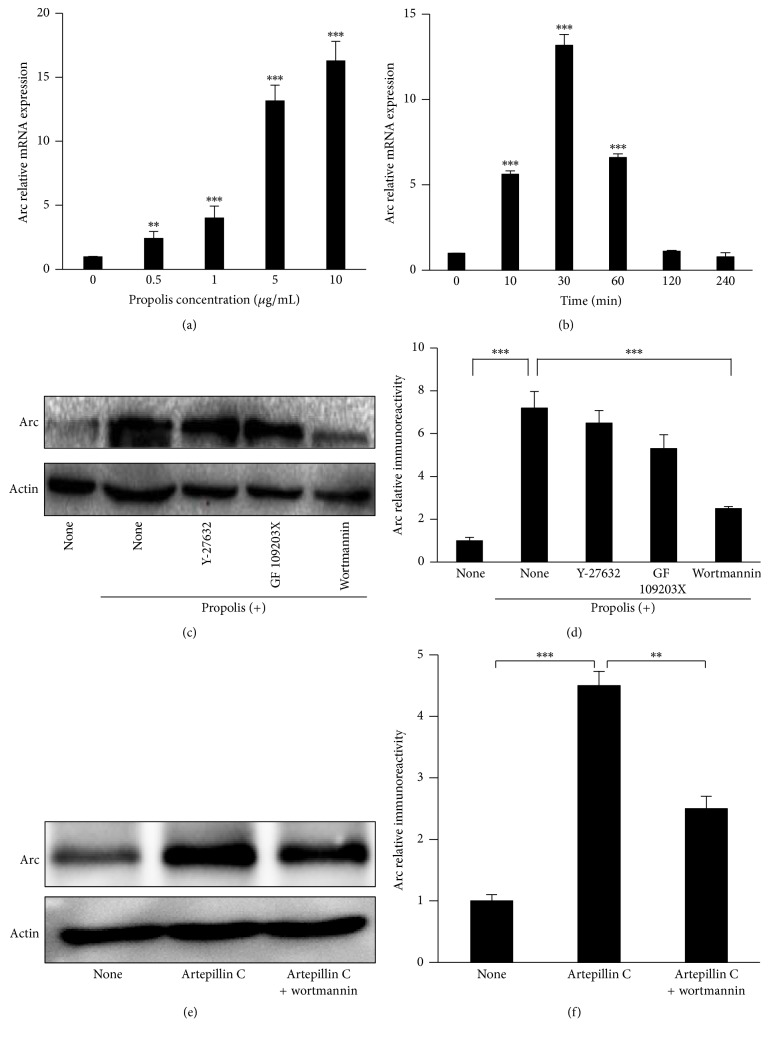
Effect of methanol extracts of propolis on Arc expression in SH-SY5Y cells. (a) Dose-dependent effects of propolis on Arc mRNA expression in SH-SY5Y cells at 30 min after incubation. (b) Time-dependent effects of 10 *μ*g/mL propolis on Arc mRNA expression in SH-SY5Y cells. (c) Effects of specific inhibitors on Arc protein expression. GF109203X hydrochloride (protein kinase C inhibitor, 200 *μ*M), Y-27632 dihydrochloride (Rho-associated protein kinase inhibitor, 1 *μ*M), and Wortmannin (phosphoinositide 3-kinase inhibitor, 200 nM) were pretreated for 1 h, followed by incubation with 10 *μ*g/mL propolis for 30 min. (d) The quantitative analyses of Arc protein expression. (e) The expression of Arc after treatment with Artepillin C (20 *μ*M) and pretreatment with Wortmannin. (f) The quantitative analysis of Arc protein expression shown in (e). The results are expressed as the mean ± SEM (*n* = 4 each), and the asterisks indicate a statistically significant difference from the control value or propolis-treated group (^*∗∗*^*p* < 0.005, ^*∗∗∗*^*p* < 0.001).

We further examined the effects of propolis on Arc expression in SH-SY5Y cells. Arc expression is very low in untreated SH-SY5Y cells. Surprisingly, even low-level (0.5 *μ*g/mL) pretreatment with propolis for 30 min significantly increased the Arc mRNA expression in SH-SY5Y cells in a dose-dependent manner. The safety dose of propolis (5 *μ*g/mL and 10 *μ*g/mL) induced Arc mRNA expression 13- and 16-fold found in untreated cultured cells (Figure 6(a)). We further investigated the time-dependent regulation of propolis (10 *μ*g/mL) on Arc expression. Arc mRNA expression is significantly increased as early as 10 min after treatment, peaking at 30 min after, lasting for 60 min, and eventually recovering to the untreated level (the basal levels) at 120 min after propolis treatment (Figure 6(b)). Our data indicated that the Arc mRNA expression-inducing effects of 10 *μ*g/mL propolis treatment peaked at 30 min; therefore, this concentration and time point were chosen for subsequent experiments.

We finally analyzed the signaling pathways of the effects of propolis on Arc induction. Pretreatment with Wortmannin (200 nM) completely abolished the propolis-induced Arc expression in SH-SY5Y cells; however, Y27632 (1 *μ*M) or GFX (200 nM) did not reduce the expression (Figure 6(c)). Furthermore, propolis (10 *μ*g/mL) for 60 min significantly increased the Arc protein expression, and the propolis-increased Arc protein expression was significantly reduced by Wortmannin, but not by Y27632 or GFX (Figures 6(c) and 6(d)). These data indicate that propolis acts through PI-3K-dependent pathways for enhancing Arc transcription and protein production.

Artepillin C, a polyphenol with a molecular weight of 300.4 extracted from Brazilian green propolis, has been reported to have the effect on neurite outgrowth of PC12 cells and the signaling pathways involved [33]. Therefore, we also analyzed the effects of Artepillin C on the expression level of Arc in SH-SY5Y cells. We found that Artepillin C significantly increased the protein expression of Arc in SH-SY5Y cells; however, the Artepillin-increased Arc was significantly reduced by pretreatment with Wortmannin. The response of Artepillin C in SH-SY5Y cells was consistent with treatment with propolis. Therefore, Artepillin C may be one of the functional components of propolis that acts through PI-3K-dependent pathways for enhancing Arc transcription and protein production.

## 4. Discussion

**Figure 7:**
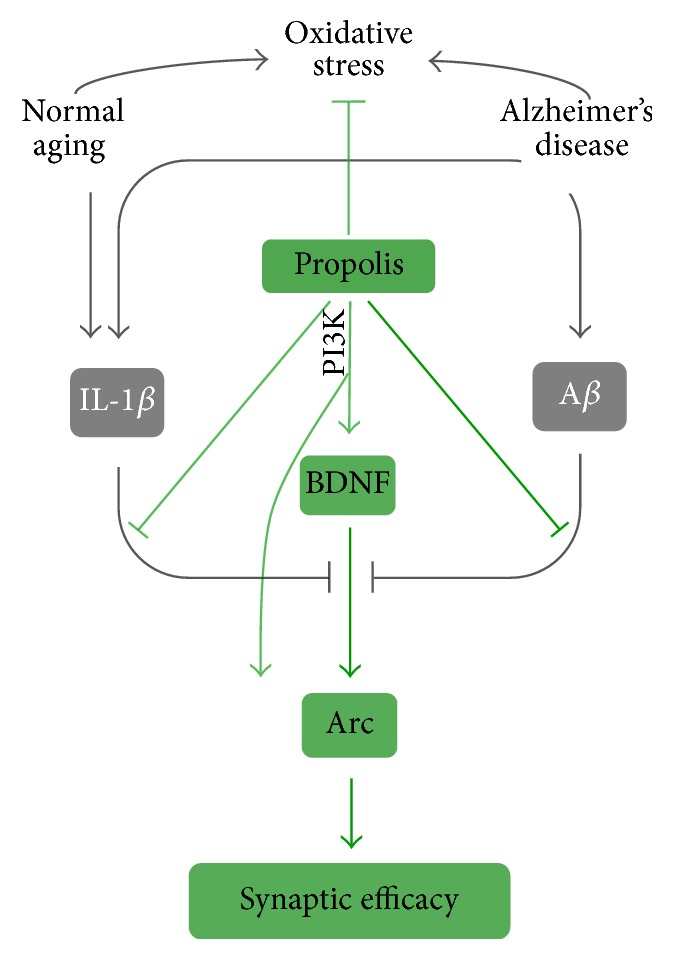
A schematic representation of the effects and the principle molecular mechanisms of Brazilian green propolis on neurodegenerative damage-induced oxidative stress and downregulation of synaptic efficacy.

The major findings of the present study are that Brazilian green propolis decreases the oxidative stress but increases the neurodegenerative dysregulated factors of synapse efficacy in human neuronal SH-SY5Y cells (summarized in Figure 7). To our knowledge, this is the first report to explore the directly neuroprotective effects of propolis on neurodegenerative damage.

Oxidative stress is a major harmful component to induce neurodegenerative damage in AD [3, 4], because oxidative stress with overproduction of ROS causes damage to the cellular components, including DNA, resulting in subsequent cell death. Antioxidant therapy therefore is considered as an approach in prevention and clinical management of AD [6]. We used an in vitro model of human neuronal SH-SY5Y cells to investigate the direct effect of Brazilian green propolis (propolis) and its signaling transductions on Arc expression to avoid other effectors. Pretreatment with propolis could protect the H_2_O_2_-induced cell death, which agreed with those of the previous reports showing baccharin, p-Coumaric acid, and Artepillin C; the active components of propolis are effective in reducing neurotoxicity [29–32]. We found that propolis significantly inhibited the H_2_O_2_-induced mitochondria ROS generation as well as nuclear DNA damage in SH-SY5Y cells (Figures 1 and 2). Thus we provide the first evidence that propolis attenuates the oxidative stress-induced DNA damage directly in neuronal cells.

The dysfunction of synaptic efficacy is an early neurodegenerative damage in AD [1, 21], because the impairment of synaptic activation precedes substantial A*β* accumulation and neuron loss in the brain [10, 41, 42]. BDNF-induced Arc expression is widely used as a marker of defective synaptic efficacy [21, 26, 43]. In the present study, we found that preexposure to fA*β* (a neurodegenerative hallmark) at a nonlethal concentration (5 *μ*M) impaired BDNF-induced Arc expression (Figure 3(b)); however, fA*β* at a nonlethal concentration did not affect Arc expression in SH-SY5Y cells (data not shown). These data were consistent with the findings of previous studies showing that BDNF-induced Arc expression was inhibited by pretreatment with oligomeric A*β* or fA*β* in cultured cortical neurons [21, 26, 44]. Surprisingly, treatment with (10 *μ*g/mL) for 120 min significantly reversed the fA*β*-induced impairment of BDNF-induced Arc expression in SH-SY5Y cells. Since a decline in Arc expression is correlated with cognitive impairment in AD [45, 46], BDNF-induced Arc expression fundamentally regulates the synaptic efficacy [17, 18]. These observations indicate that propolis can prevent the fA*β*-induced dysfunction of synaptic efficacy in neuronal cells.

Increasing evidence indicates that microglia-related neuroinflammation contributes to the decline in cognitive function during aging as well as in AD [47, 48]. As a potent activator for exacerbating neuroinflammation, IL-1*β* has been reported to suppress BDNF-dependent synaptic efficacy, resulting in cognitive impairment [26, 49]. Furthermore, the overexpression of IL-1*β* in a transgenic mouse model resulted in increased microglia activation with a significant reduction in behaviorally induced Arc levels and impaired contextual and spatial memory [50]. In the present study, we demonstrated that nonlethal concentrations of IL-1*β* (20 ng/mL) impaired BDNF-induced Arc expression in SH-SY5Y cells (Figures 4(b)–4(d)), a finding that agreed with those of a previous report showing that IL-1*β* suppressed the BDNF-induced Arc expression in cultured brain slices [26]. Importantly, treatment with propolis significantly reversed the IL-1*β*-induced impairment of BDNF-induced Arc expression. These observations indicate that propolis can reverse the neuroinflammation-induced dysfunction of synaptic efficacy in neuronal cells.

BDNF, as a neurotropic factor, fundamentally controls the synaptic efficacy [17, 51] and closely correlates with cognitive functions [14, 19]. Indeed, BDNF levels are reduced even in the preclinical stages of AD [52]. In the present study, treatment with propolis significantly increased the BDNF expression in SH-SY5Y cells in dose- and time-dependent manners. Furthermore, the propolis-upregulated BNDF expression was completely abolished by preincubation with PI3K inhibitor, indicating that the effects of propolis on BDNF expression are mediated by the PI3K signaling pathway.

Arc expression was low in the untreated SH-SY5Y cells, which agreed with the findings of other reports using cultured primary cortical neurons [53, 54]. Exogenous treatment with propolis induced Arc expression in SH-SY5Y cells in both dose- and time-dependent manners. Of note, exposure to propolis induced rapid, robust Arc mRNA expression, as quickly as 10 min after exposure and peaking at 30 min, which paralleled the Arc expression peaks around 30 min after behavior induction [55]. Therefore, Arc may serve as an early beginning effector molecule for propolis-induced neuronal activity.

The PI3K signaling pathway plays a pivotal role in synaptic efficacy and memory consolidation [56–58]. The observations, of which the effects of propolis on Arc expression are mediated by the PI-3K signaling pathway, thus provide the underlying mechanism of propolis in directly regulating the molecule for synaptic efficacy in human neuronal cells. Artepillin C, a major component of Brazilian green propolis, has also been found to act as a neurotrophic-like factor for promoting NGF-induced neurite outgrowth [33]. Our ongoing human research at high altitude shows that elderly individuals take propolis score significantly higher on cognitive tests than nontreated individuals (Wu & Zhu et al., unpublished data). The neuroprotective effects of Brazilian green propolis on neurodegenerative damage might provide a valuable therapeutic strategy for prevention of cognitive impairment in AD as well as aging.

## 5. Conclusion

Brazilian green propolis could reduce oxidative stress and prevent the neurodegenerative damaged synapse efficacy (schematic represented in Figure 7). Therefore, we provide the principle molecular mechanisms of the benefits of propolis as the therapeutic agent for maintenance cognitive function of brain.
